# Novel organic draw solution in forward osmosis process for fertigation: performance evaluation and flux prediction

**DOI:** 10.1007/s11356-022-20674-4

**Published:** 2022-05-12

**Authors:** Ghada Al Bazedi, Noha Soliman, Hani Sewilam

**Affiliations:** 1 Center for Applied Research on the Environment and Sustainability (CARES), School of Science and Engineering, The American University in Cairo, AUC Avenue, P.O. Box: 74, New Cairo, 11835 Egypt; 2 Department of Engineering Hydrology, RWTH Aachen University, Mies-van-der-Rohe Strasse 17, 52074 Aachen, Germany; 3 Chemical Engineering & Pilot Plant Department, Engineering Research Division, National Research Center, 33 El-Bohouth St, Dokki, 12311 Cairo Post Code Egypt

**Keywords:** Forward osmosis, Organic fertilizer, Performance, Spirulina, Flux

## Abstract

Fertilizer-drawn forward osmosis (FDFO) has received a lot of attention for its potential for producing fertigated water for agriculture purposes. To minimize the use of chemical-based fertilizers and support sustainable organic agriculture, this work investigated the separation performance of FO membrane for different feed concentrations (FS) of brackish water using microalgae *Spirulina platensis* as an organic fertilizer draw solution (DS). Different feed solution concentrations were investigated ranging 3–20 g/L NaCl, with various draw solutions of spirulina ranging 280–440 g/L. The performance was measured by water flux and recovery. The results showed that using spirulina as a draw solution is a promising solution for fertigation purposes. The results showed that Na^+^ in feed solution is concentrated by 41%, Cl^-^ by 36%, and spirulina is diluted by 20% for feed salinity 5000 mg/L. The highest flux obtained with different feed solution 3000/5000/10,000/20,000 mg/L were 9/6/4.5/7 for draw solution concentration of 360/360/400/420 g/L. The calculated specific reverse solute flux (SRSF) *J*_S_/*J*_W_ varies from 0.1 and 0.8 for different explored FS/DS concentrations. Flux decline and the down-time was investigated for the highest flux observed, showing 290 min of operation before cleaning action is required.

## Introduction

Water scarcity is an emerging global crisis becoming more complex due to growing populations, fast development, and climate change. The most efficient approach to address the issue of water scarcity is water desalination (Boretti and Rosa; 2019, Mancosu, et al; 2015). The most common challenges of desalination are the high energy consumption and the negative environmental impact of brine disposal. Forward osmosis (FO) has lately reinvigorated consideration as a low-energy desalination method. FO process is used for various applications including both brackish (Giagnorio et al. 2019) and seawater desalination, wastewater treatment, food industry, and biomass concentrations (Hoover et al. 2011). Forward osmosis is being utilized to desalinate brackish water, which is a novel field of research. One of its uses is fertilizer-drawn forward osmosis (FDFO), which provides a feasible alternative water supply for irrigation. Using this approach in the context of the water-energy-food nexus is very promising for addressing water scarcity problem for food production with minimum energy while avoiding any trade-off with other sustainability pillars. When compared to conventional desalination methods, the FDFO desalination technique enhances the supply of agricultural-quality water suitable for crop growth while using significantly less energy. Because FDFO is a low-energy technology, it may be powered by renewable energy, making it adaptable and versatile for a wide range of distant applications (Nasr and Sewilam 2015; Amin et al. 2021).

In the FDFO system, the draw solution (DS) is performed from fertilizers, while brackish/seawater serves as a feed solution. A fertigation distribution network is then used to apply the diluted fertilizer solution to crops. According to Zou and He (2016), 1 kg of fertilizer may extract 2459 L of freshwater from high salinity synthesized brackish water.

FDFO offers a great potential in many FO applications due to their relatively higher water flow rate and absence of recovery process requirements. The DS and the membrane characteristics have a significant impact on the performance of the FO process. Therefore, choosing the right DS is critical for process efficiency (Haupt and Lerch 2018).

Previous research studies aimed on investigating the use of forward osmosis in desalination of either brackish water or seawater, where the produced water is directly used for fertigation without recovery process for the DS. Inorganic-based compounds have been used as the DS in the majority of FO investigations, and they are still extensively employed. Different studies by Phuntsho et al. (2011, 2012, and 2013) showed that utilizing fertilizers as a DS for direct fertigation processes is promising. Nasr and Sewilam (2015) have illustrated the use of FDFO for brackish water treatment, also El Zayat (2021) evaluated the concentration of artificial brine using a FDFO method utilizing an industrial-grade (NH_4_).2SO_4_. The observed flux was 11.69 L/h/m^2^ when the DS used was (NH_4_).2SO_4_ with artificial brine as FS.

Electrolyte solutions form the majority of inorganic-based which are used as DS; however, non-electrolyte solutions are also applicable (Long, Q. 2018, El Zayat 2019; Tan et al. 2010). Tayel et al. (2020) experimented the use of uncoated magnetic nanoparticles (MNPs) as a DS for FO desalination process. The results showed that MNPs as a DS have a substantial effect on the flux of the produced water. A mixture from organic and metal salt was investigated by Nematzadeh et al. (2020), where they explored the usage of blended amino-acids–metal-salts fertilizer DS and their performances through forward osmosis process. Caspian Sea water desalination was also experimented showing the effectiveness of using an arginine–ZnCl2 fertilizer as a draw solution.

Different integrated systems comprising FDFO and microfiltration (MF)/ultrafiltration (UF) or nanofiltration (NF) have been used to optimize the operation of the FDFO system (Phuntsho et al.2013b). Kim et al. (2017) tested and compared an integrated FDFO/NF hybrid model with standard reverse osmosis (RO) and with hybrid scenarios employing MF or UF as a pre-treatment procedure. The results showed that the integrated FDFO/NF system employing a thin-film composite forward osmosis (TFC-FO) membrane had lower energy consumption compared to other studied integrated systems.

Chemical-based fertilizers have been used extensively to fulfil the need for high nutritional deficit in the soil. The regular use of chemical-based fertilizers had an impact on crop quality, as well as soil health. Fertilizers derived from microalgae have recently been shown to be superior to chemical-based fertilizers, not only because they include potassium, phosphorus, and nitrogen, but also because they are high in metabolites and plant growth regulators such as auxins, cytokinin, and gibberellins (Deepika and MubarakAli 2020; Kumar and Baweja 2012, 2018). Organic fertilizer/bio-fertilizer is also appropriate due to its cost-effectiveness and mitigation of harmful effects of synthetic fertilizers, as well as ecological efficiency (Shaji et al. 2021; Dahama et al. 2003).

The microalga *Spirulina platensis* was investigated as a potential fertilizer (Faheed et al. 2008; Aung et al. 2011; Dineshkumar et al. 2018; Sridhar and Rengasamy 2002). Wuang et al. (2016) studied using spirulina as an agricultural fertilizer when coupled with aquaculture wastewater. The results showed a significant improvement in the growth of leafy vegetables and in the seedling dry weight. Spirulina is also used in some studies to reduce soil erosion and pollution caused by the application of chemical fertilizers, as well as increasing the soil fertility (Anitha et al. 2020).

The main purpose of this study is to explore the efficiency of the separation performance of FO membrane for different feed concentrations (FS) by using as organic fertilizer the microalgae *Spirulina platensis* as DS.

## Materials and methods

### Membrane setup

**Figure 1.**
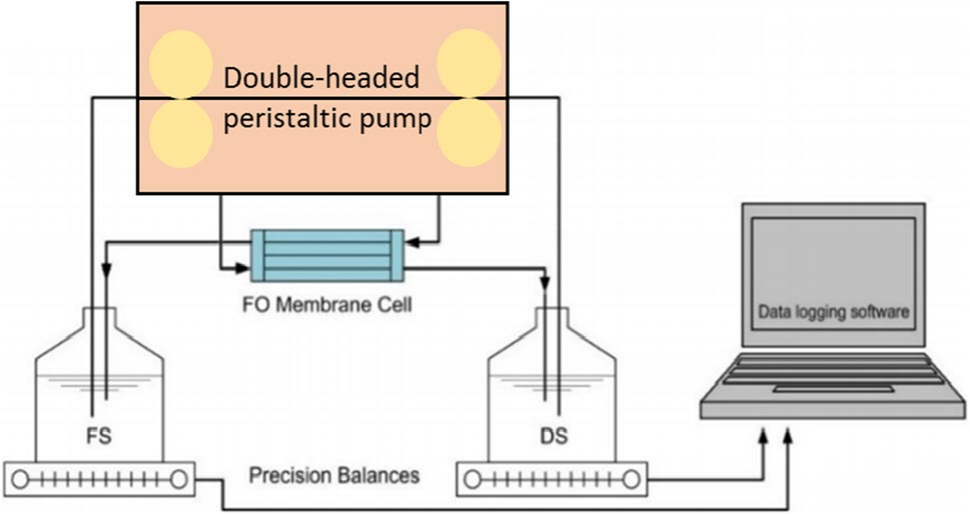
Experimental setup

The setups for the experiments were performed using a bench-scale crossflow unit as presented in Fig. 1. The FO unit comprises a membrane with diameter of 40 mm, an area of 1.257 × 10^-3^ m^2^, and an average flow of 200 mL/min, as presented in Table 1. FO mode of operation is used along the experiments. FS is facing the membrane active layer and the DS is facing the support layer. The membrane and the fluxometer were produced by Porifera Inc., USA.

**Table 1:** Porifera’s FO membrane operating guidelines. Modified from Porifera Inc. (2016) and Nasr (2016)

Item	Specifications
Pure water permeability coefficient, *A* (Lm-2 h-1 bar-1)	2.2 ± 0.01
Salt permeability coefficient of active layer, *B* (m/s)	1.6 × 10.7
Total membrane thickness (μm)	70 ± 10
Material of active layer	Polyamide (PA)
Material of support layer	Porous hydrophilic polymer
Water permeation	FO mode: 33 ± 2 LMH
Membrane parameters structural parameter	(*S* value): 215 ± 30 μm
Maximum trans-membrane pressure (TMP)	180 psi
pH operating range	2–11
Maximum chlorine	Chlorine < 0.1 mg/L

### Organic fertilizer

In the current investigation, *Spirulina platensis* are photosynthetic, multicellular blue green microalgae that thrive in a variety of fresh, marine, and brackish water environments (Marrez et al. 2014). One kilogram of fresh algal material was chopped into tiny pieces and weighed. A mixer was used to extract the sample. The blended mixture was filtered through a double-layered muslin cloth to eliminate debris and labeled as 100%. The different concentrations employed in this investigation were made by adding DI water (Pise and Sabale 2010). The source of fresh algae is Algal Biotechnology Unit, NRC, Egypt.

The organic fertilizer draw solution (OFDS), comprising mainly spirulina, was prepared with different concentrations ranging 240–480 g/L. The characteristics of the organic DS are presented in Table 2.

**Table 2:** Composition of organic fertilizer

Component	Wt.%
Spirulina	96.35
CaO	0.4
MgO	0.25
K_2_O	1.5
P_2_O_5_	1.5

### Chemicals

Sodium chloride (NaCl, 99%), analytical grade purchased from ADWIC Pharmaceutical and Chemicals Company, Egypt, was used in the experiments. The feed solution was prepared by dissolving the salt in DI water with uniform mixing using a magnetic stirrer at 200–300 rpm to ensure that all salts are dissolved. All tests were carried out at room temperature.

### Methods

All experiments are performed at ambient temperature. Water flux was calculated by the change in feed solution volume in the feed solution tank. Online digital recording of data has been used to register any change in water volume continuously at 2-min time intervals using a digital precision balances (Mettler Toledo Precision Balances XS4002S) to give real-time measurements of the change in mass at preset intervals. The water flux *J*_w_ (in L/m^2^/h) is calculated using the following equation.

1 $$Jw=\Delta V/(A*(T))$$

where, ∆V Total volume to the DS from FS

*A* Membrane area

*T* Time

Both the DS and the FS had a starting mass of 125 g. Along the process, DS was constantly diluted as the feed concentration increased, which caused flow rate reduction with time. The water flux, on the other hand, was detected from the flux/time plot, where the steady flow occurred within the first 15 min of operation. The tests lasted at least 2 to 3 h to allow adequate DS diffusion and good monitoring of any reverse DS diffusion.

Reverse salt permeation is undesirable to FO because it not only disrupts feed water concentration control and decreases the net osmotic driving force, but it also raises the fouling tendency of the FS by forming complexes with the feed ions. Dependent on the solute characteristics, reverse solute flow varies dramatically for each fertilizer. It should be observed that DS containing ions with high hydrated diameters had less reverse permeability than DS containing ions with lower hydrated diameters (Zhao et al. 2012, Nasr and Sewilam 2016). Because of the concentration difference, reverse permeation or reverse diffusion of the solute from the DS to the FS is predicted.

2 $$RSF=Js (Vi-\Delta V)*Cs/ membrane area$$

where:

Vi Stands for the primary volume of FS

ΔVStands for the water volume transferred from FS to the DS

Cs Stands for the concentration of the draw solute in the FS after experiment

The osmolality (Osmol/kg) of draw solutions at various concentrations was measured using an osmometer (Osmomat 030, cryoscopic osmometer, Gonotec) at Cairo University. Using Eq. (3), the osmolality of draw solutions was converted to osmotic pressure (atm) at a temperature of (22°C ±1°C).

3 $$OP=RTc$$

where, OP (atm) is the osmotic pressure. RT (kg · atm/mol) = 24.22 at 22°C. And *c* (moles/kg) is the draw solution osmolality.

## Results and discussion

Different FS concentrations (3000–20,000 mg/L) were selected for these experiments, where the pH of the FS solution ranges from 6.0 to 6.5. These concentrations were selected as representative of different brackish groundwater salinity in Egypt. Five concentrations of the selected spirulina organic fertilizer DS ranging from 240 to 480 g/L were investigated, where the pH of the DS ranges from 5 to 5.5.

The DS osmotic pressure has been measured using an osmometer as presented in Table 3. Figures 4, 5, 6, and 7 show the flux for different DS concentrations vs different FS concentrations. Higher DS concentration shows higher flux owing to higher osmotic pressure of the solution. Lower flux is detected at low DS concentration of 280 g/L.

**Table 3:** Osmotic pressure of different DS concentrations

DS concentration, g/L	280	320	360	400	420	480
Osmotic pressure (bar)	10	12	13	15	15.4	17

### Baseline investigations

**Figure 2:**
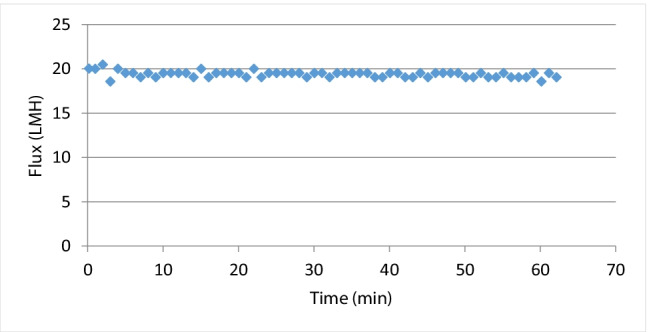
Baseline experiments, water flux with 1 M NaCl as DS

**Figure 3:**
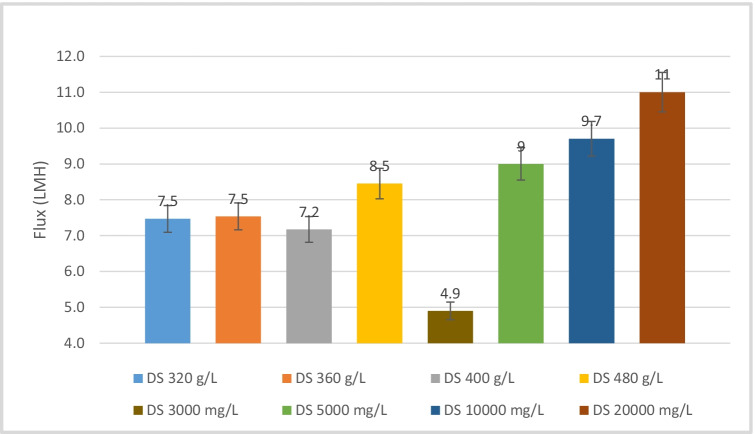
Flux determination for OFDS different concentrations (320–480 g/L) and NaCl different concentrations (3000–20,000 mg/L) as DS, DI as FS

Baseline experiments (Figures 2, 3) were performed to investigate the flux rate of the membrane process. The first was DI water as FS and 1M of NaCl as DS. The second experiment was DI as FS and DS with different concentrations of spirulina organic fertilizer (OFDS) ranging from 240 to 480 g/L. The third investigation was done to determine the flux of different NaCl concentrations as DS and DI as FS. Total dissolved solids (TDS) were measured by a portable TDS and EC meter (Orion Thermo Scientific) before and after each experiment. The membrane was cleaned after each experiment for the same FS concentration values using inline DI cleaning. All the experiments were carried out in AL-FS mode, where the active layer is facing the feed solution. The first baseline experiments were performed to assess the membrane’s fundamental functionality and to provide baseline measurements for the produced water flow using DI water as the FS.

The first baseline experiment was carried out after each cleaning run to evaluate if membrane cleaning was adequate for recovering membrane performance and how repeated usage affected membrane performance. As long as there was no membrane damage found during the membrane performance evaluation, the same membrane was utilized again after a complete washing with DI water.

The second baseline experiment was carried out to investigate and identify the flux of different selected concentrations of the organic fertilizer in DI. The highest water flux was observed at DS concentration of 480 g/L as shown in Figure 3. Then, the DI was replaced with different feed concentrations of analytical grade NaCl (3, 5, 10, 20 g/L). Also, investigating the flux for different DS concentrations of NaCl (3000–20,000 mg/L) and DI as FS.

### ODFO performance evaluation

**Figure 4:**
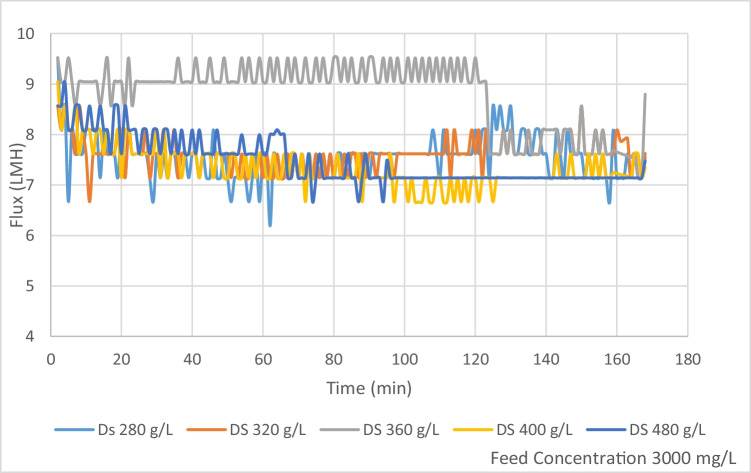
Flux for different DS for FS TDS 3000 mg/L

The investigation was conducted to evaluate the efficiency and separation performance of using organic fertilizer of the microalgae *Spirulina platensis* as the draw solution (OFDS) for different feed concentrations representing different brackish water concentrations in Egypt. The first FS concentration investigated in this study was set at 3000 mg/L. As shown in Figure 4, DS concentration of 360 g/L showed the higher flux of an average 9 L/(m^2^·h) for the first 120 min of operation, then a sudden decline in flux to an average of 7.8 L/(m^2^·h) afterwards. DS concentration of 480 g/L showed a fixed flux rate of 7.14 L/(m^2^·h) after 95 min of operation, while DS 400 g/L showed a sudden decline in flux to 6.66 L/(m^2^·h) after 100 min of operation to rise again to 7.14 L/(m^2^·h) after 25 min of lower flux interval.

**Figure 5:**
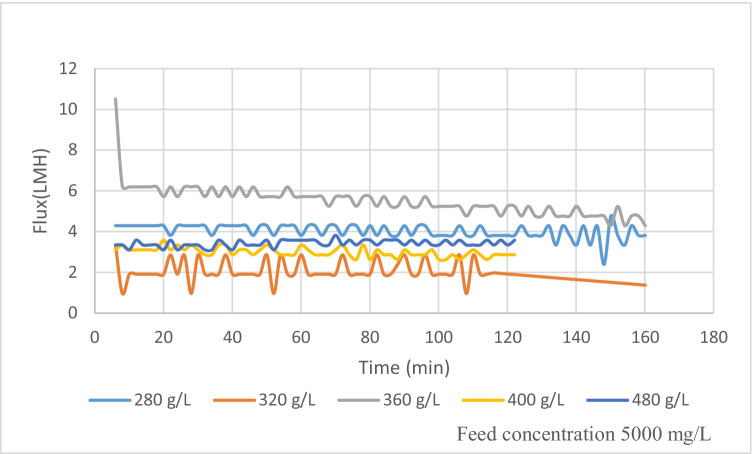
Flux for different DS for FS TDS 5000 mg/L

**Figure 6:**
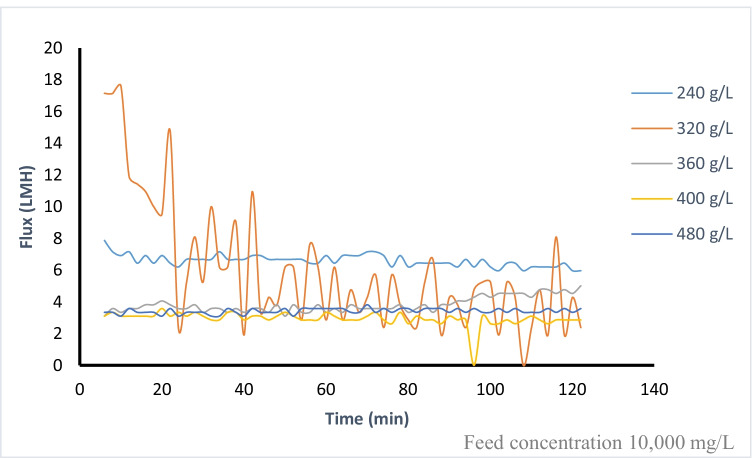
Flux for different DS for FS 10,000 mg/L

It was observed as presented in Figure 5, for feed concentration 5000 mg/L, that better flux is obtained when increasing the concentration of the DS. At draw solution of concentration 360 g/L, the corresponding flux reaches 5.2 LMH at the end of the experiment. The water flux increases from 1.6 to 5.2 L/(m^2^·h) as presented in Figure 6 after 150 min of operation for DS concentration from 280 to 360 g/L, while for DS concentrations of 400 and 480 g/L, the flux ranges between 3.3 and 3.5 L/(m^2^·h). This is attributed to the building up of foulants atop the membrane surface due to the high concentration of organic matter.

The water flux increases from 2.8 to 3.5 L/(m^2^·h) as presented in Figure 6, when increasing the draw solution concentration from 280 to 360 g/L for feed solution concentration of 10,000 mg/L. Concentration polarization CP effect is responsible for the non-linear flow behavior as DS concentrations increase. This low flux is attributed to the low pressure difference between FS and DS solutions. When compared to other experiments utilizing inorganic fertilizers, the observed flux is much lower than that of inorganic DS using the same type of membrane adopted by Nasr and Sewilam (2015). However, comparing the obtained results with other organic DS shows a good agreement with the experimental results by Wang et al. (2017) who used a tailor-made TFC membrane, and the results obtained by Alaswad et al. (2018) who used a nanofiltration membrane (TFC-SR2) in FS-AL mode for investigating the performance of sucrose and glucose as draw solutions against deionized water in a forward osmosis (FO). In another comparison with the obtained results using other organic draw solutions as presented in Islam et al. (2019), the reported flux is higher due to higher osmotic pressure differences between feed and draw solutions. Due to its low viscosity and high solubility, Gwak et al. (2015) demonstrated that the DS solution with the lowest molecular weight created the greatest water flow. Also, because of their comparatively high osmotic pressure and big molecule sizes, carboxyethyl amine sodium salts were utilized as draw solutes, resulting in a substantially larger water flow and a reduced reverse solute flux in PRO mode (Long et al. 2015). The flux and reverse permeation of the draw solute from the DS side to the FS side have a significant impact on the efficiency of the FDFO process.

**Figure 7:**
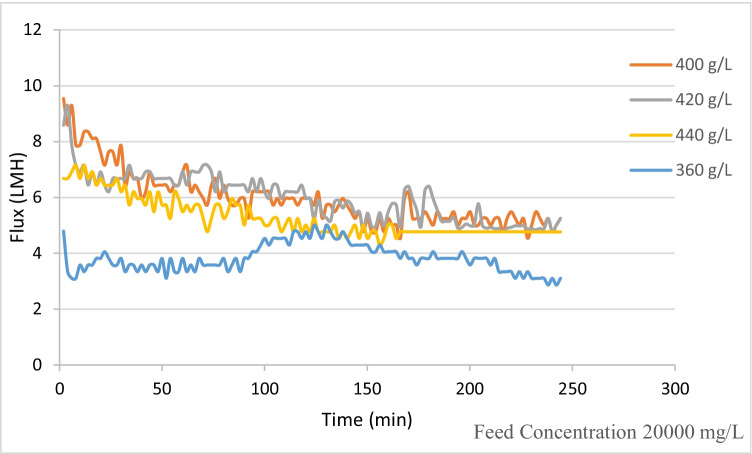
Flux for different DS for FS 20,000 mg/L

**Figure 8:**
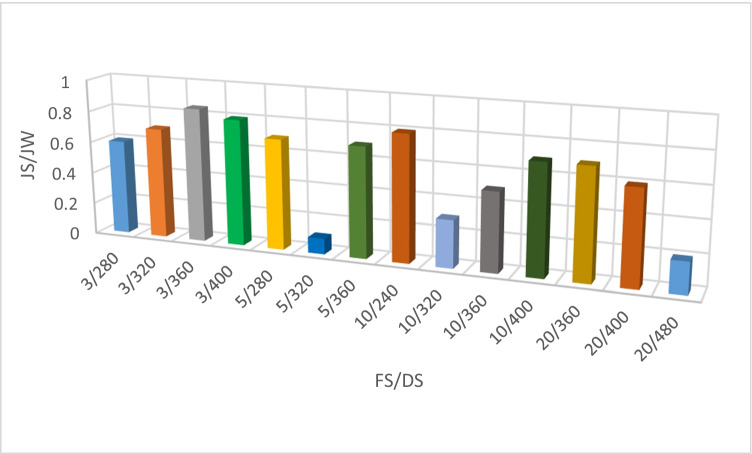
Membrane selectivity *J*_S_/*J*_W_ for different FS/DS concentrations

Figure 7 shows that the water flux increased from 2.8 to 5.2 L/(m^2^·h) by the end of the experiment on increasing the draw solution concentration from 360 to 440 g/L for feed solution concentration of 20,000 mg/L. The flux patterns for draw solution concentration 400 and 420 g/L show a similar pathway in the first 50 min of operation. A slight difference in flux values was indicated afterwards. Reverse permeation from DS was not detected due to the formation of the organic fouling layer. Specific reverse solute flux (SRSF) *J*_S_/*J*_W_ values are much higher at fluxes less than 10 Lm^-2^h^-1^ than at fluxes more than 10 Lm^-2^h^-1^. Membrane selectivity *J*_S_/*J*_W_ was calculated for different feed and DS concentrations, as presented in Figure 8. The calculated *J*_S_/*J*_W_ varies from 0.1 and 0.8 for the various DS concentrations; this is due to the fact that increased DS concentrations result in more severe ICP effect. Wang et al. (2017) detected draw solutes with high specific reverse salt flow while employing polyepoxysuccinic acid. Bowden et al. (2012) employed organic draw solutes in FO with a high SRSF of 0.65 g/L. Some of the examined organic draw solutes had higher solubility and osmotic pressure than inorganic draw solutes, but more crucially, all of the organic draw solutes evaluated had lower specific reverse salt fluxes.

Sanahuja-Embuena et al. (2019) reported that HF–C membranes have a *J*_s_/*J*_w_ = 0.4–0.1 g/L for inorganic DS. The primary explanation for the greater *J*_s_/*J*_w_ of the HF–C membranes is an increase in the salt permeation coefficient. While Keng et al. (2021) investigated PEG-400 specific RSF in acetone showing 0.1877 g/L, particular RSF is an intrinsic characteristic of the selective layer; this shows that the membrane’s selectivity altered in various organic solvents.

### Down-time of membrane performance

**Figure 9:**
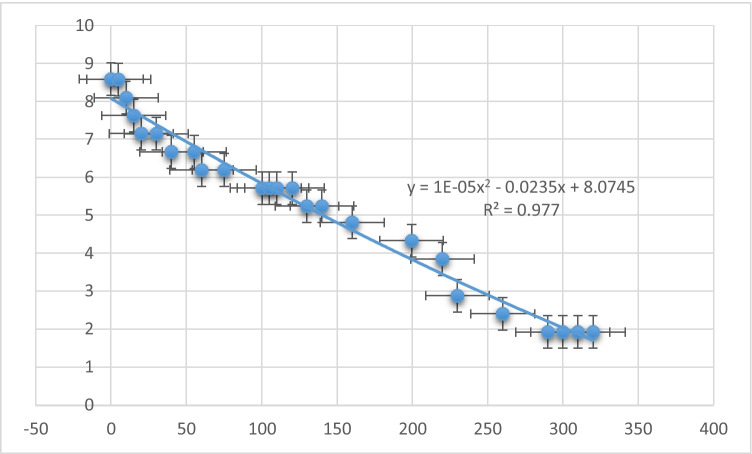
Down-time for ODFO (feed 3000 mg/L)

By exploring the flux decline and the down-time required for membrane cleaning at the highest flux observed through all the experiments, the maximum flux was obtained for FS 3000 mg/L and DS 360 g/L, and the flux decline experiment was duplicated to ensure the results. The down-time was reached after 290 min of operation. The performance of the membrane shows different flux decline phases while operating: the first was after 125 min of operation and the second was after 150 min as shown in Figure 9. The last flux decline phase was observed at the period of 230–290 min of operation to reach a min value of flux of 1.9 L/(m^2^·h). The reduced pressure was differential across the membrane surface, resulting in reduced water flow causing flux decline. Furthermore, the ICP on the membrane surface resulted in limited water flow (Alaswad et al. 2018). The flux decline pattern shows a nonlinear relation with coefficient of determination (*R*^2^) of 0.977.

When comparing the results to the published organic draw solute flux values, it is clear that the flux is within the reported range by Al-Alalawy (2017) and Alaswad et al. (2018). It can be concluded that the organic DS exhibit low diffusion coefficients that are affected by ICP causing lower flux compared to that reported for inorganic DS. The higher flux values would be attributable to a decrease in the external concentration polarization effect at both membrane interfaces.

A significant effect of applying highly concentrated DS is the need of a special pump that can tolerate the high viscosity of the DS. This selection is controlled by fluid characteristics including the type of solution, viscosity, etc. (Phuntsho et al. 2012).

**Figure 10:**
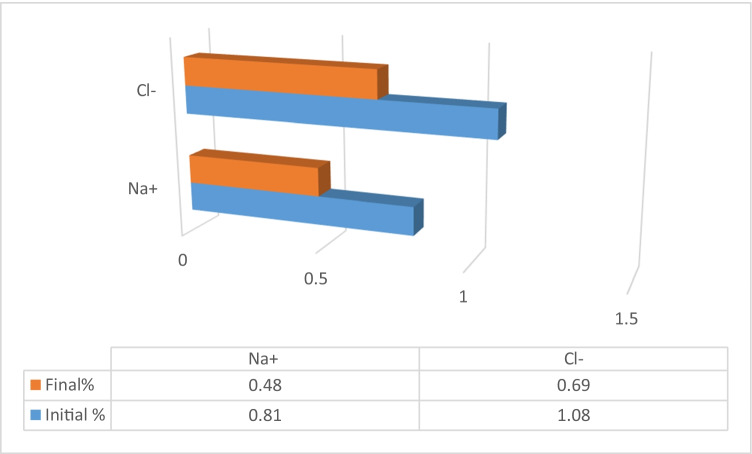
Feed solution initial and final concentrations (feed 3000–5000 mg/L)

**Figure 11:**
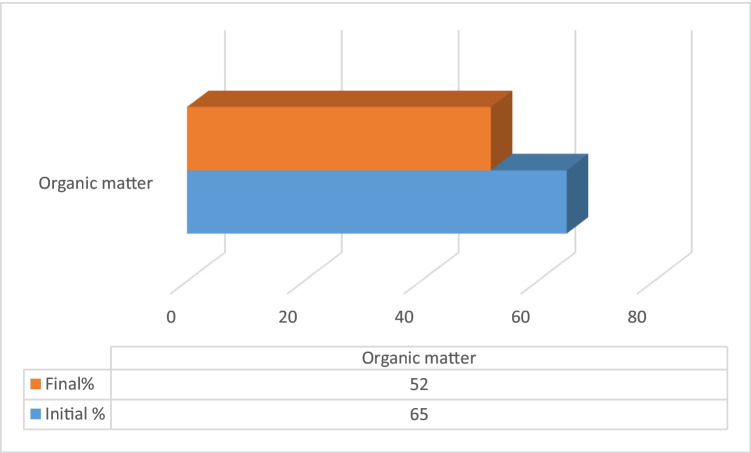
Draw solution initial and final organic content concentrations for (feed 3000–5000 mg/L)

The changes in feed and draw solution concentration were calculated by analyzing the final concentration of each. Figures 10 and 11 present the initial and final concentrations for 5000 mg/L feed solution.

### Multivariate regression analysis

This part focuses on the construction of a simple empirical model for estimating membrane performance (flux) by specifying the concentration of OFDS. The flux shows a non-linear relation with DS concentration. By applying multivariate regression analysis for experimental results and using the average flux for each feed concentration, the flux estimation equations are as follows for the required range of feed water quality. A regression model with coefficient of determination (*R*^2^) of 1 for FS salinities 3000–5000 mg/L, while *R*^2^ of 0.85 and 0.99 for FS salinities 10,000–20,000 mg/L. The model was verified with a 15% average variance for higher FS concentration and essentially no deviation for lower FS concentrations.

For feed water of salinity 3000 mg/L, the *R*^2^ for the estimated flux is 1. The flux estimation equation shows a polynomial 4^th^ order equation.

$${\varvec{F}}{\varvec{l}}{\varvec{u}}{\varvec{x}}= = 0.3294{\varvec{x}}^4 - 3.9145{\varvec{x}}^3 + 15.799{\varvec{x}}^2 - 24.856{\varvec{x}} + 20.189$$

For feed water of salinity 5000 mg/L, the *R*^2^ for the estimated flux is 1. The flux estimation equation shows a polynomial 6^th^ order equation.

$${\varvec{F}}{\varvec{l}}{\varvec{u}}{\varvec{x}}=-2{\varvec{E}}-05{\varvec{x}}^3 + 0.0188{\varvec{x}}^2 - 6.23{\varvec{x}} + 684.9$$

For feed water of salinity 10,000 mg/L, the *R*^2^ for the estimated flux is 0.85.

$${\varvec{F}}{\varvec{l}}{\varvec{u}}{\varvec{x}}=0.0001{\varvec{x}}2 - 0.1252{\varvec{x}} + 28.659$$

The non-linear flux behavior is due to high concentration of DS which causes unfavorable effects, like increasing the concentration polarization effect leading to further flux decline behavior (Boyu Liu et al. 2015).

For feed water of salinity 20,000 mg/L, the *R*^2^ for the estimated flux is 0.99.

$${\varvec{F}}{\varvec{l}}{\varvec{u}}{\varvec{x}}== 0.0063{\varvec{x}} + 1.75$$

Where,

*x* Spirulina concentration, g/L

The results showed that by increasing the DS concentration, there is a significant rise in flux. A flux decline was observed after 90 min of operation, which is due to rapid cake formation on the membrane surface.

## Conclusion

The microalga *Spirulina* is a low-cost economic organic fertilizer that can be used as a DS in forward osmosis system. In this study, we examine the microalgae *Spirulina platensis* as a novel organic fertilizer for organic fertilizer-drawn forward osmosis (ODFO), and the efficiency of the separation performance of FO membrane for different feed concentrations (FS) by using organic fertilizer microalgae *Spirulina platensis* as draw solution (DS).

By using the microalgae *Spirulina platensis* as a draw solution, the water permeation flux (water flux) was measured, and the average percent dilution of DS was investigated. As a result of the performance evaluation, the following conclusions were drawn.

The osmotic pressure of the microalgae *Spirulina platensis* draw solution concentrations ranges between 10 and 15.4 bar

The percent changes in concentration for feed and draw solution were investigated as Na^+^ in feed solution is concentrated by 41% and Cl^-^ by 36%, and spirulina is diluted by 20% for feed salinity 3000–5000 mg/L

Microalgae *Spirulina platensis* show a promising draw solution for FDFO process intended for fertigation purposes

The major problem was the nonlinear flux behavior in low feed salinity concentrations due to concentration polarization, which needs to be investigated further.

Reverse permeation from DS solution was not detected which is due to the difficulties in obtaining precise measurements/analysis of solute permeation from the draw to the feed solution and the formation of the organic fouling layer.

The main aim of the process is to use an organic fertilizer to reduce the harm of chemical fertilizers as well as increasing the soil fertility. The diluted DS solution can be used as a fertilizer after further dilution according to the plant requirements or type of application (seeds soaking in *Spirulina*/*Spirulina* in combination with biofertilizers, chemical fertilizer, and vermicompost/foliar spray) (Godlewska et al. 2019).

The results of this study clearly demonstrate that using spirulina as draw solution for FDFO has some good potential for practical use. However, investigations of FO membranes at the module level are still restricted; thus, a more comprehensive research of fouling tendencies and effective cleaning techniques is required. More research should be performed based on the findings of this study to focus on module configurations and pressure performance at the module level of the FO process so as to improve and get a more precise, reliable evaluation of the FDFO process.

## Data Availability

The datasets used and/or analyzed during the current study are available from the corresponding author on reasonable request.
